# Synthesis and structure of *N*-(perfluoro­phen­yl)isonicotinamide

**DOI:** 10.1107/S2056989025010679

**Published:** 2026-01-01

**Authors:** Arindam Saha, Garry S Hanan, Mihaela Cibian

**Affiliations:** a Département de chimie, Université de Montréal, Complexe des sciences, 1375, Avenue Thérèse-Lavoie-Roux, Montréal, Québec, H2V 0B3, Canada; b Département de biochimie, chimie, physique et science forensique and l’Institut de recherche sur l’hydrogène, Université du Québec à Trois-Rivières, 3351, boul. des Forges, CP 500, Trois-Rivières, Québec, G9A 5H7, Canada; University of Aberdeen, United Kingdom

**Keywords:** crystal structure, aryl­amide, hydrogen bonding

## Abstract

The title compound [systematic name: *N*-(2,3,4,5,6-penta­fluoro­phen­yl)pyridine-4-carboxamide], C_12_H_5_F_5_N_2_O, crystallizes with two independent mol­ecules (*A* and *B*) in the asymmetric unit in space group *P*1. The mol­ecules adopt a conformation where the planes of the penta­fluoro­phenyl and pyridyl rings have twist angles of 5.3 (1) and 14.5 (1)°. In the crystal, the mol­ecules are connected by N—H⋯N hydrogen bonds between the amide H atom and the pyridyl nitro­gen atom of an adjacent mol­ecule to generate [110] chains of alternating *A* and *B* mol­ecules. The packing is consolidated by C—H⋯π inter­actions, π–π stacking and C—H⋯O inter­actions and a short F⋯F contact of 2.7270 (13) Å occurs. The structures of related isonicotinamides are surveyed.

## Abstract 2

The title compound crystallizes with two independent mol­ecules in the asymmetric unit, which are connected into chains by N—H⋯O hydrogen bonds.

## 1. Chemical context

There is ongoing inter­est in the synthesis of amides for academic and industrial research (Pattabiraman & Bode, 2011 ▸) owing to their applications in peptide synthesis (Seward & Jakubke, 2002 ▸), drug discovery (Masse *et al.*, 1998 ▸), organo­metallic (Leitch *et al.*, 2011 ▸), and coordination chemistry (Hasegawa *et al.*, 2007 ▸). Herein, we report on the synthesis and solid state structure of the title compound, C_12_H_5_F_5_N_2_O (**1**). It was sythesized as the first-step product of a total three-step method for preparing amidine-*N*-oxide ligands (Cibian *et al.*, 2009 ▸, 2011 ▸; Saha *et al.*, 2024 ▸). Although there are specific examples in the literature corresponding to unsymmetrical amides, this is the first report of pyridyl-containing N—H penta­fluroaryl analogue.
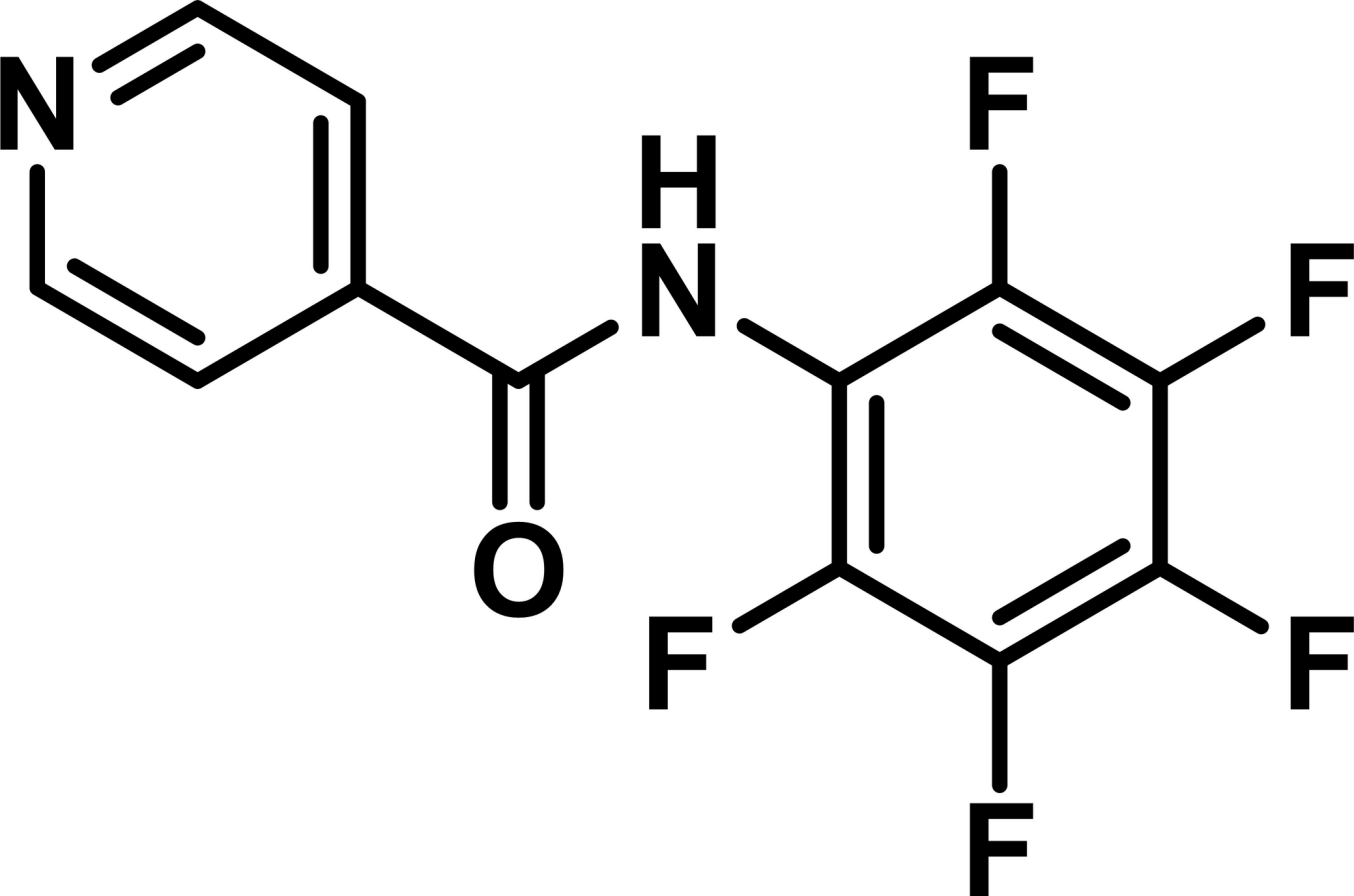


## 2. Structural commentary

**Figure 1:**
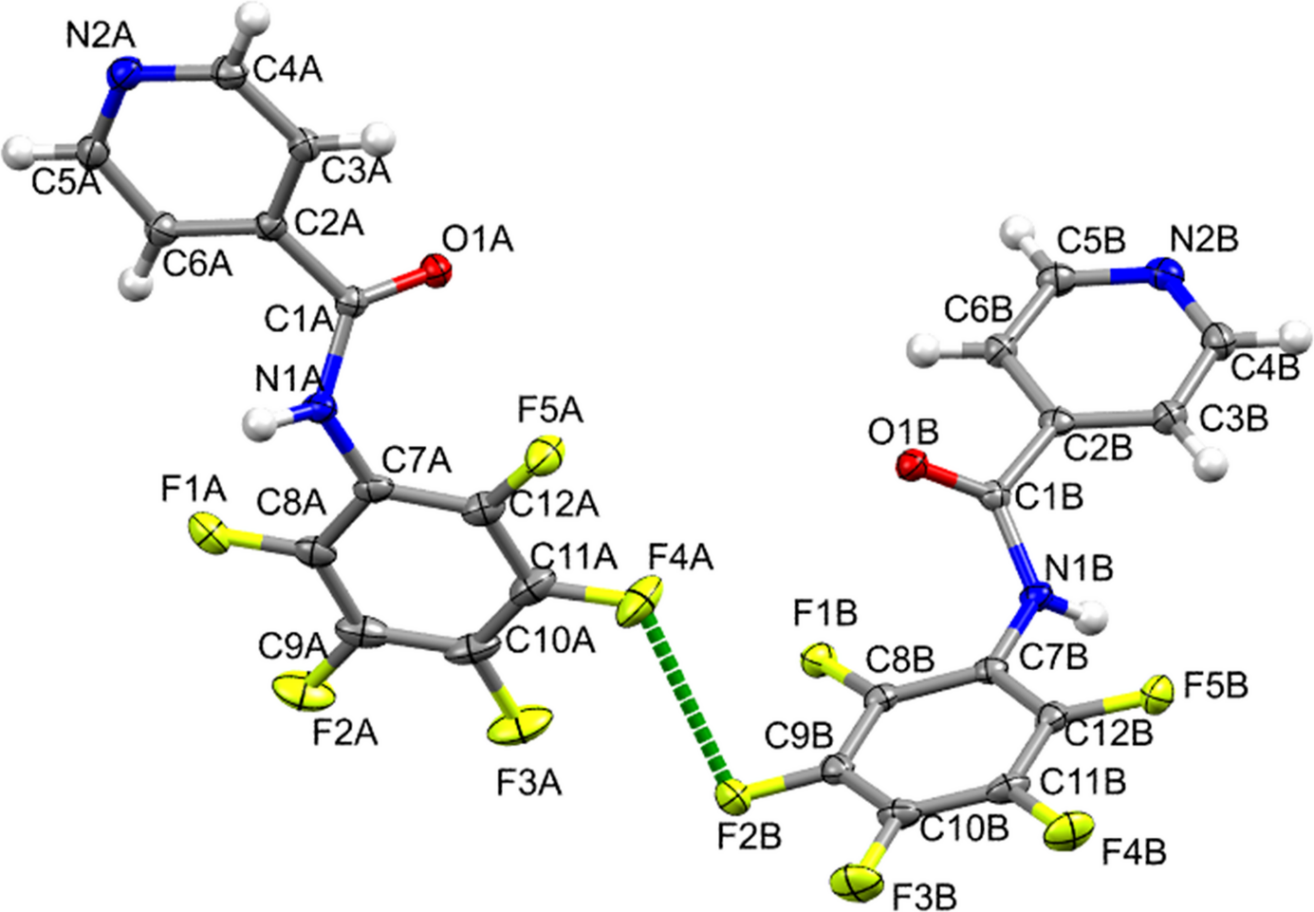
The mol­ecular structure of **1**, with displacement ellipsoids drawn at the 50% probability level. The short F⋯F inter­molecular contact is also shown.

Each of the two mol­ecules in the asymmetric unit of **1** consists of three near planar sub-units: the 4-pyridyl ring (py), the amide linkage (NCO), and the penta­fluoro­phenyl ring (pfp) (Fig. 1 ▸). Tilt angles exist between the py and pfp planes. In mol­ecule *A* (atoms labelled with the suffix *A*), this twist angle has a value of 5.3 (1)° whereas in mol­ecule *B* (atoms labelled with the suffix *B*) this value is 14.5 (1)°. In mol­ecule *A*, the angles between the amide plane (consisting of atoms N1*A*, C1*A*, and O1*A*) and the py and pfp planes are 47.8 (1) and 49.7 (1)°, respectively. Regarding mol­ecule *B*, these values (plane of amide consisting of atoms N1*B*, C1*B*, and O1*B* with the py and pfp rings) are 38.5 (1) and 52.8 (1)°. The N1*A*—C1*A* and N1*B*—C1*B* bond lengths [indistinguishable within 3σ, average of 1.360 (1) Å] are characteristic of the partial double-bond found in amides. This inter­mediate length, falling between that of a typical N—C single bond (∼1.45) Å and an N=C double bond (∼1.25 Å), results from resonance across the amide group (Pattabiraman & Bode, 2011 ▸). The amide N—C bond length herein is statistically similar (within 3σ) to those found in other *N*-(penta­fluoro­phen­yl)aryl­amides with the aryl group being phenyl, 4-nitro­phenyl, or 4-di­methyl­amino­phenyl [1.369 (4), 1.369 (5), and 1.366 (4) Å, respectively, Adams *et al.*, 2001 ▸], but it is different when the aryl substituent is another penta­fluoro­phenyl ring [1.332 (5) Å; Pagliari *et al.*, 2022 ▸]. The amide N—C bond length in **1** is also similar to those found in *N*-aryl-substituted isonicotinamides bearing *N*-substituents such as phenyl [1.359 (2) Å; Mondal *et al.*, 2007 ▸] or 4-fluoro­phenyl [1.355 (2) Å; Mocilac *et al.*, 2011 ▸]. However, it is quite different when a bulky *N*-aryl-substituent is present, *e.g*., 2,6-di*iP*rPh [1.337 (1) Å; Laramée *et al.*, 2012 ▸]. The amide C=O bond length in **1** is similar to that in *N*-(phen­yl)penta­fluoro­benzamide [1.232 (4) Å; Adams *et al.*, 2001 ▸], but it is longer in *N*-(penta­fluoro­phen­yl)penta­fluoro­benzamide [1.271 (4) Å; Pagliari *et al.*, 2022 ▸], as expected due to the electron-withdrawing effect of the penta­fluoro­phenyl substituent. The amide C=O bond length in **1** is also shorter than those observed in *N*-aryl-substituted isonicotinamides bearing *N*-substituents such as phenyl [1.232 (2) Å; Mondal *et al.*, 2007 ▸], 4-fluoro­phenyl [1.230 (2) Å; Mocilac *et al.*, 2011 ▸], and 2,6-di*iP*rPh [1.333 (1) Å; Laramée *et al.*, 2012 ▸].

## 3. Supra­molecular features

**Figure 2:**
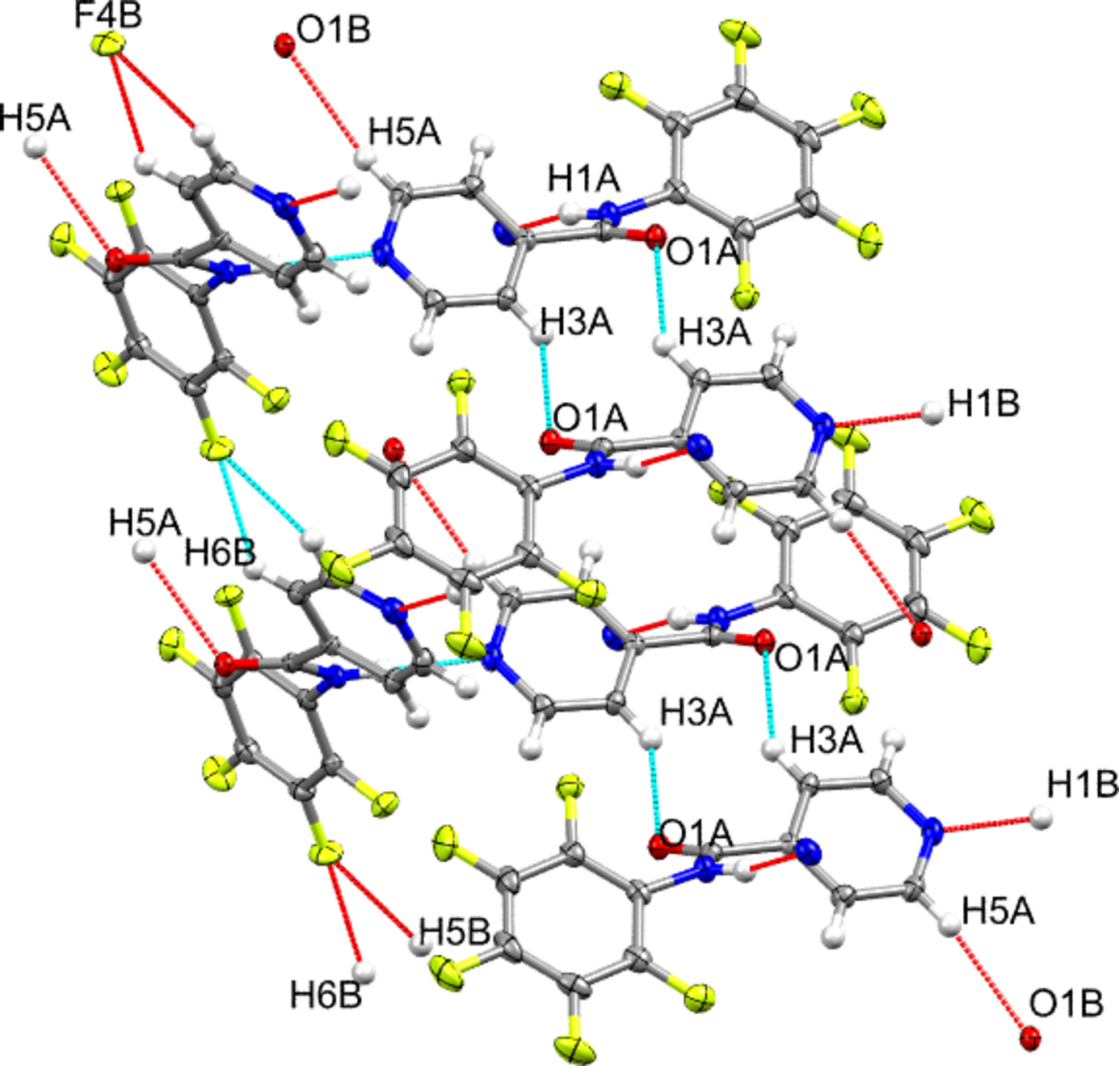
Hydrogen-bonding pattern in **1**.

**Figure 3:**
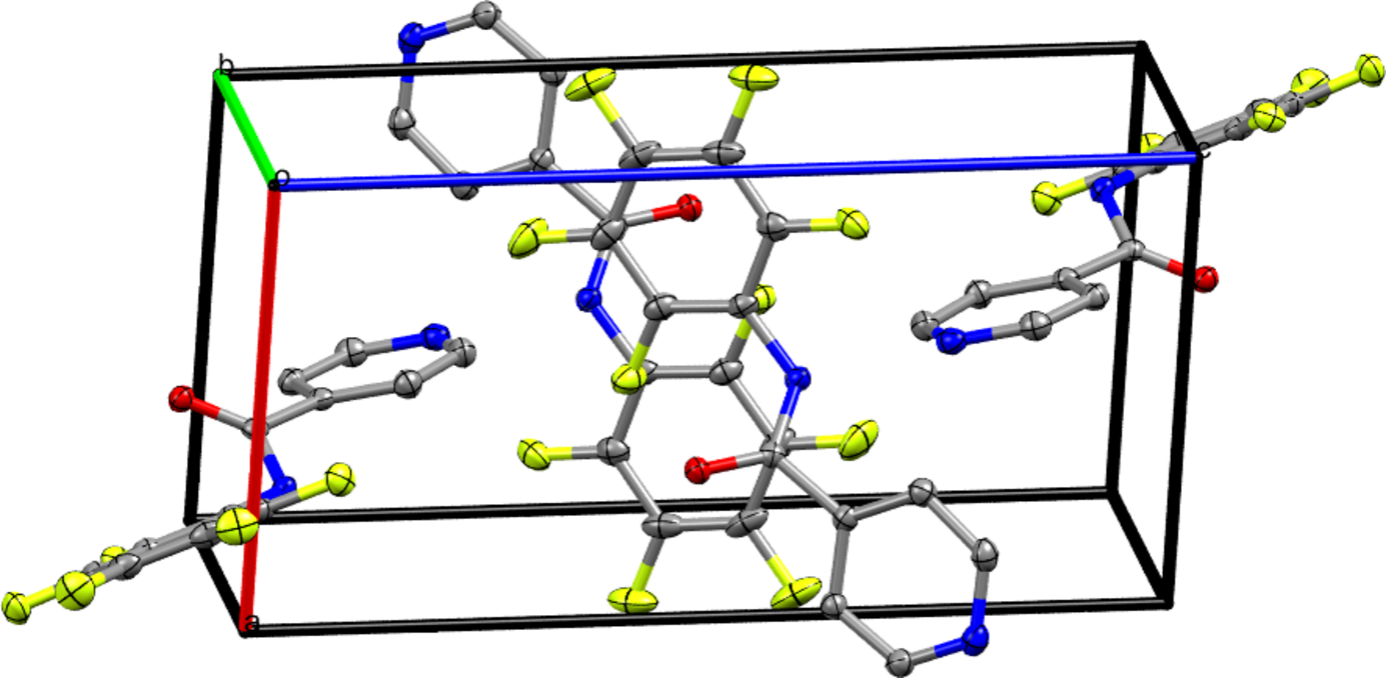
Packing of **1** in the unit cell. H atoms are omitted for clarity.

In the crystal, the mol­ecules are linked by N1*A*—H1*A*⋯N2*B* and N1*B*—H1*B*⋯N2A hydrogen bonds (Table 1 ▸ and Fig. 2 ▸) between the amide (donor) and py subunits (acceptor), generating [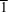
10] chains of alternating *A* and *B* mol­ecules. Given the short distance between the donor and acceptor units with a high hydrogen-bond angle, these inter­actions are likely to be strong (Desiraju & Steiner, 2001 ▸). Hydrogen-bonding inter­actions also exist between the *o-*C*sp*^2^–H (C3*A*—H3*A* in *A*) and the carbonyl-O atom (O1*A* in *A*) located in two adjacent unit cells. These inter­actions are assigned as moderately weak hydrogen bonds. In contrast, the hydrogen bond between *m-*C*sp*^2^–H (C5*A*—H5*A* in *A*) and carbonyl-O (O1*B* in *B*) is stronger, given a C5*A*—H5*A*⋯O1*B* distance of 2.54 (1) Å and angle of 169.8 (1)°. Inter­estingly, the fluorine atom in mol­ecule *B* (F4*B* in the pfp ring) is also engaged as a double acceptor with two *sp*^2^C—H atoms (H5*B* and H6*B* in mol­ecule *B*) in the adjacent mol­ecule. The bond parameters for C5B—H5*B*⋯F4*B* are 2.65 (1) Å and 117 (1)° and those for C6*B*—H6*B*⋯F4*B* are 2.43 (1) Å and 124 (1)°. This observation could also explain the higher twist angle between the py and pfp planes in mol­ecule *B* where the F atom is involved in the above-mentioned inter­actions, which are absent in mol­ecule *A*. The packing in **1** (Fig. 3 ▸) is further consolidated by C—H ⋯π inter­actions and π–π stacking inter­actions, as well as by a short F4*A*⋯F2*B* contact (shown in Fig. 1 ▸) of 2.7270 (13) Å (van der Waals sum = 2.94 Å). This C—F⋯F—C inter­action is identified as quasi type I/II (Singla *et al.*, 2023 ▸).

## 4. Database survey

Table 2 ▸ presents the results of the Cambridge Structural Database survey with respect to other reported mol­ecular structures of *N*-(ar­yl)isonicotinamides and *N*-(perfluoro­phen­yl)aryl­amides (CSD, Version 5.46, update of November 2024; Groom *et al.*, 2016 ▸). The space groups and the values of tilt angle between the aromatic rings (θ) are presented for each of the structures. All the compounds in Table 2 ▸ are free amides [*N*-(ar­yl)isonicotinamides (entries 1–14) and *N*-(perfluoro­phen­yl)aryl­amides (entries 20–27) or pyridinium chloride salts (entries 15–19)], non-coordinated to metal ions. Coordination complexes of related amide ligands containing 4-py subunit exist with transition-metal ions Cu^II^ (CSD refcodes FOPZAM, FOPZEQ, FOPZIU; Ge *et al.*, 2005 ▸ and JEQMEY, JESXUB; Ge *et al.*, 2006 ▸), Cd^II^ (IKEQIY; Li *et al.*, 2003 ▸), and Zn^II^ (IKEQOE; Li *et al.*, 2003 ▸ and QINJEF, QINJIJ; Kwiatek *et al.*, 2019 ▸). Mono- and bimetallic 4-py amide-based coordination polymers are reported with Cu^II^ (IDUTIN; Chen *et al.*, 2018 ▸, and ISISUZ; Deng *et al.*, 2011 ▸) and Co^III^ (IPURIV; Chen *et al.*, 2011*a* ▸), Cu^II^/Tb^III^ (NETWOA; Deng *et al.*, 2013 ▸), Cu^II^/Gd^III^ (IZAYIS; Chen *et al.*, 2011*b* ▸), Mn^II^/Gd^III^ (NASMIF; Chen *et al.*, 2012 ▸), and Mn^II^/Eu^III^ (NASMEB; Chen *et al.*, 2012 ▸).

Several coordination polymers based on discrete units containing 4-py O-/S- linked bis­amides are also reported with transition-metal ions such as Mn^II^ (JEMMOG, JEMPOJ, JEMQOK), Ni^II^ (JEMQAW, JEMQIE), and Co^II^ (JEMQEA, JEMPUP) (Tzeng *et al.*, 2016 ▸). Inter­estingly, 2,3,4,5,6-penta­fluoro-*N*-(penta­fluoro­phen­yl)benzamide and *N*-phenyl­benzamide are also reported as co-crystallized structures (RENPEJ; Pagliari *et al.*, 2022 ▸).

Other *N*-(R)isonicotinamides exists, with *R* = Me (PAPROP; Mukherjee *et al.*, 2011 ▸), as well as *N*-(perfluoro­phen­yl)-R*-*amides, with *R* = Me (WALPIL; Babailov *et al.*, 2015 ▸), CF_3_ (TEKQOP; Mahoui *et al.*, 1996 ▸), di­meth­oxy­phosphinoyl (XIPNEQ and XIPPOC; Song *et al.*, 2007 ▸), and other more exotic groups [DORKUR (Moorthy *et al.*, 2009 ▸), DIJGAF, DIJJAI, DIJJEM (Basheer *et al.*, 2007 ▸)]. *N*-(Perfluoro­phen­yl)-*R*-bis­amides (AMEKAG, AMEKAG01, NIPXUG; Light *et al.*, 2016 ▸, Picci *et al.*, 2020 ▸, and Light *et al.*, 2008 ▸) are also reported.

## 5. Synthesis and crystallization

The synthesis of compound **1** was realized by reacting isonicotinic acid (0.30 g, 2.44 mmol, 1 equiv.) and penta­fluoro­aniline (1.12 g, 6.10 mmol, 2.5 equiv.) in polyphospho­ric acid tri­methyl­silyl ester (PPSE) at 453 K, overnight. The reaction was brought to room temperature and quenched with aqueous NaOH 1 *M*. A beige solid was obtained, which was further recrystallized from 95% aqueous EtOH solution and dried under vacuum to give the pure compound, as colorless crystals. Yield 0.49 g, 70%. The final product was characterized by ^1^H and ^19^F NMR, as well as by C/H/N elemental analysis. Note: The PPSE (a condensing and dehydrating agent) was obtained as a colorless viscous liquid by refluxing P_2_O_5_ with hexa­methyl­disiloxane (HMDS) (stoichiometry 1 to 1.5) in dry DCM for 30 min (under N_2_), followed by solvent evaporation.

## 6. Refinement

Crystal data, data collection and structure refinement details are summarized in Table 3 ▸. The H atoms were included in calculated positions and treated as riding atoms: aromatic C—H = 0.95 Å, methyl C—H = 0.98 Å, with *U*_iso_(H) = *k* × *U*_eq_(parent C atom), where *k* = 1.2 for the aromatic H atoms and 1.5 for the methyl H atoms. The amide H atoms (H1A and H1*B*) were located in a difference-Fourier map and refined freely.

## Supplementary Material

Crystal structure: contains datablock(s) I. DOI: 10.1107/S2056989025010679/hb8158sup1.cif

Supporting information file. DOI: 10.1107/S2056989025010679/hb8158Isup3.cml

Structure factors: contains datablock(s) I. DOI: 10.1107/S2056989025010679/hb8158Isup3.hkl

CCDC reference: 2512078

Additional supporting information:  crystallographic information; 3D view; checkCIF report

Additional supporting information:  crystallographic information; 3D view; checkCIF report

## Figures and Tables

**Table 1:** Hydrogen-bond geometry (Å, °)

*D*—H⋯*A*	*D*—H	H⋯*A*	*D*⋯*A*	*D*—H⋯*A*
N1*A*—H1*A*⋯N2*B*^i^	0.872 (18)	2.004 (19)	2.8261 (16)	156.9 (16)
N1*B*—H1*B*⋯N2*A*^ii^	0.844 (18)	2.047 (19)	2.8643 (16)	163.1 (17)
C3*A*—H3*A*⋯O1*A*^iii^	0.95	2.44	3.2075 (17)	138
C5*A*—H5*A*⋯O1*B*^iv^	0.95	2.54	3.4785 (17)	170
C6*B*—H6*B*⋯F4*B*^v^	0.95	2.43	3.0669 (16)	124

Symmetry codes: (i) 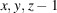; (ii) 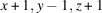; (iii) 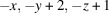; (iv) 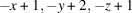; (v) 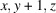.

**Table 2:** CSD reported mol­ecular structures of *N*-(ar­yl)isonicotinamides and *N*-(perfluoro­phen­yl)aryl­amides (free base, non-coordinated and salts forms) py = pyridyl; pfp = penta­fluoro­phenyl; Ar1 = 3-(meth­oxy­carbon­yl)-2-methyl­phenyl; Ar2 = 3-(meth­oxy­carbon­yl)-6-methyl­phenyl; Ar3 = 5-(meth­oxy­carbon­yl)-2-methyl­phenyl; Ar4 = 2-(meth­oxy­carbon­yl)-4-methyl­phenyl; Ar5 = 4-(meth­oxy­carbon­yl)-2-methyl­phenyl; Ar6 = 9-anthracene; Ar7 = 4-di­methyl­amino­phenyl; Ar8 = 4-nitro­phenyl; Ar9 = 5′-methyl, 2′-meth­oxy-biphenyl-4-carboxyl­ate; Ar10 = 4-fluoro-2-methyl-6-(morpholin-4-yl); θ = tilt angle between the aromatic rings.

Entry No.	*R*1—(C=O)	*R*2—(N—C=O)	Space group	θ (°)	CSD refcode	Reference
1	4-py	Ph	*P* 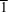	63	PEDDIM	Kumar *et al.* (2004 ▸)
2	4-py	Ph	*P* 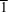	61	PEDDIM01	Mondal *et al.* (2007 ▸)
3	4-py	Ph	*P* 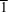	61	PEDDIM02	Mondal *et al.* (2020 ▸)
4	4-py	4-F—Ph	*P* 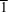	58	AMUDES	Mocilac *et al.* (2011 ▸)
5	4-py	3-F—Ph	*Cc*	66	AMUDIW	Mocilac *et al.* (2011 ▸)
6	4-py	3-F—Ph	P21/c	69	KODGES	Mocilac *et al.* (2018 ▸)
7	4-py	2-F—Ph	*Cc*	77	AMUDOC	Mocilac *et al.* (2011 ▸)
8	4-py	2,6-diiPr-Ph	P21/c	80	CEGMOS	Laramée *et al.* (2012 ▸)
9	4-py	4-Cl—Ph	Pbca	48	KEHTOK	Gallagher *et al.* (2022 ▸)
10	4-py	3-Cl—Ph	P21/n	2	KEHTUQ	Gallagher *et al.* (2022 ▸)
11	4-py	2-Cl—Ph	*Cc*	83	KEHVAY	Gallagher *et al.* (2022 ▸)
12	4-py	4-MePh	P2/c	67	UXEXAX	Mocilac *et al.* (2011 ▸)
13	4-py	3-MePh	P21/n	5	UXEXEB	Mocilac *et al.* (2011 ▸)
14	4-py	2-MePh	*Cc*	84	UXEXIF	Mocilac *et al.* (2011 ▸)
15	4-pyH^+^	Ar1	P21/c	10	DAZGAP	Kwiatek *et al.* (2017 ▸)
16	4-pyH^+^	Ar2	P21/c	88	DAZFOC	Kwiatek *et al.* (2017 ▸)
17	4-pyH^+^	Ar3	P41	88	DAZGET	Kwiatek *et al.* (2017 ▸)
18	4-pyH^+^	Ar4	P21/c	13	QINKEG	Kwiatek *et al.* (2019 ▸)
19	4-pyH^+^	Ar5	P21/c	4	QINKIK	Kwiatek *et al.* (2019 ▸)
20	Ar6	pfp	P21/n	2, 58	CABGAO	Adams *et al.* (2001 ▸)
21	Ar7	pfp	P21/n	22	UCOVAJ	Adams *et al.* (2001 ▸)
22	Ar8	pfp	*Cc*	81	UCOVEN	Adams *et al.* (2001 ▸)
23	Ar9	pfp	P21/c	3	AKUDIV	Wang *et al.* (2016 ▸)
24	Ar10	pfp	*C*2/*c*	38	VODGEE	Xing *et al.* (2023 ▸)
25	pfp	pfp	P1	86	RENPAF	Pagliari *et al.* (2022 ▸)
26	pfp	pfp	P21/c	90	QUKVUN	Sopkova *et al.* (2001 ▸)
27	pfp	pfp	*P* 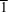	89	QUKVUN01	Adams *et al.* (2001 ▸)

**Table 3:** Experimental details

Crystal data	
Chemical formula	C_12_H_5_F_5_N_2_O
*M*_r_	288.18
Crystal system, space group	Triclinic, *P* 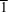
Temperature (K)	100
*a*, *b*, *c* (Å)	7.6987 (1), 10.5767 (1), 14.9123 (2)
α, β, γ (°)	76.250 (1), 86.487 (1), 71.941 (1)
*V* (Å^3^)	1121.26 (2)
*Z*	4
Radiation type	Cu *K*α
μ (mm^−1^)	1.51
Crystal size (mm)	0.11 × 0.08 × 0.02

Data collection	
Diffractometer	Bruker SMART APEXII area detector
Absorption correction	Multi-scan (*SADABS*; Krause *et al.*, 2015 ▸
*T*_min_, *T*_max_	0.642, 0.753
No. of measured, independent and observed [*I* > 2σ(*I*)] reflections	31582, 4197, 3961
*R*_int_	0.020
(sin θ/λ)_max_ (Å^−1^)	0.614

Refinement	
*R*[*F*^2^ > 2σ(*F*^2^)], *wR*(*F*^2^), *S*	0.031, 0.083, 1.04
No. of reflections	4197
No. of parameters	369
H-atom treatment	H atoms treated by a mixture of independent and constrained refinement
Δρ_max_, Δρ_min_ (e Å^−3^)	0.29, −0.25

Computer programs: *APEX2* and *SAINT* (Bruker, 2009 ▸), *SHELXT* (Sheldrick, 2015*a* ▸), *SHELXL* (Sheldrick, 2015*b* ▸), *OLEX2* (Dolomanov *et al.*, 2009 ▸), *OLEX2* (Dolomanov *et al.*, 2009 ▸), *ORTEP-3 for Windows* (Farrugia, 2012 ▸), *publCIF* (Westrip, 2010 ▸), *POVRAY* (POVRAY, 2013 ▸), *PLATON* (Spek, 2020 ▸) and *Mercury* (Macrae *et al.*, 2020 ▸).

## supplementary crystallographic information

### N-(2,3,4,5,6-Pentafluorophenyl)pyridine-4-carboxamide Crystal data

**Table:**

C_12_H_5_F_5_N_2_O	*Z* = 4
*M**_r_* = 288.18	*F*(000) = 576
Triclinic, *P*1	*D*_x_ = 1.707 Mg m^−^^3^
*a* = 7.6987 (1) Å	Cu *K*α radiation, λ = 1.54178 Å
*b* = 10.5767 (1) Å	Cell parameters from 9964 reflections
*c* = 14.9123 (2) Å	θ = 3.1–70.7°
α = 76.250 (1)°	µ = 1.51 mm^−^^1^
β = 86.487 (1)°	*T* = 100 K
γ = 71.941 (1)°	Plate, colourless
*V* = 1121.26 (2) Å^3^	0.11 × 0.08 × 0.02 mm

### N-(2,3,4,5,6-Pentafluorophenyl)pyridine-4-carboxamide Data collection

**Table:**

Bruker SMART APEXII area detector diffractometer	4197 independent reflections
Radiation source: microfocus sealed X-ray tube, Incoatec Iµs	3961 reflections with *I* > 2σ(*I*)
Mirror optics monochromator	*R*_int_ = 0.020
Detector resolution: 7.9 pixels mm^-1^	θ_max_ = 71.1°, θ_min_ = 3.1°
φ and ω scans	*h* = −9→9
Absorption correction: multi-scan (SADABS; Krause *et al.*, 2015	*k* = −12→12
*T*_min_ = 0.642, *T*_max_ = 0.753	*l* = −18→18
31582 measured reflections	

### N-(2,3,4,5,6-Pentafluorophenyl)pyridine-4-carboxamide Refinement

**Table:**

Refinement on *F*^2^	Primary atom site location: iterative
Least-squares matrix: full	Hydrogen site location: mixed
*R*[*F*^2^ > 2σ(*F*^2^)] = 0.031	H atoms treated by a mixture of independent and constrained refinement
*wR*(*F*^2^) = 0.083	*w* = 1/[σ^2^(*F*_o_^2^) + (0.0443*P*)^2^ + 0.555*P*] where *P* = (*F*_o_^2^ + 2*F*_c_^2^)/3
*S* = 1.04	(Δ/σ)_max_ = 0.001
4197 reflections	Δρ_max_ = 0.29 e Å^−^^3^
369 parameters	Δρ_min_ = −0.25 e Å^−^^3^
0 restraints	

### N-(2,3,4,5,6-Pentafluorophenyl)pyridine-4-carboxamide Special details

**Table:**

Experimental. X-ray crystallographic data for 1 were collected from a crystal sample, which was mounted on a loop fiber. Data were collected using a Bruker smart diffractometer equipped with an APEX II CCD Detector, a Incoatec IMuS source and a Quazar MX mirror. The crystal-to-detector distance was 4.0 cm, and the data collection was carried out in 512 x 512 pixel mode. The initial unit cell parameters were determined by a least-squares fit of the angular setting of strong reflections, collected by a 180.0 degree scan in 180 frames over three different parts of the reciprocal space.
Geometry. All esds (except the esd in the dihedral angle between two l.s. planes) are estimated using the full covariance matrix. The cell esds are taken into account individually in the estimation of esds in distances, angles and torsion angles; correlations between esds in cell parameters are only used when they are defined by crystal symmetry. An approximate (isotropic) treatment of cell esds is used for estimating esds involving l.s. planes.

### N-(2,3,4,5,6-Pentafluorophenyl)pyridine-4-carboxamide Fractional atomic coordinates and isotropic or equivalent isotropic displacement parameters (Å^2^)

**Table:**

	*x*	*y*	*z*	*U*_iso_*/*U*_eq_	
F1A	0.83834 (11)	0.85339 (9)	0.35948 (6)	0.02830 (19)	
F2A	1.13400 (12)	0.69644 (10)	0.46658 (7)	0.0403 (2)	
F3A	1.08948 (13)	0.53557 (10)	0.63288 (8)	0.0430 (3)	
F4A	0.74626 (14)	0.53090 (9)	0.68912 (7)	0.0415 (2)	
F5A	0.45820 (11)	0.67183 (8)	0.57895 (6)	0.0291 (2)	
O1A	0.31847 (12)	0.95308 (9)	0.51675 (6)	0.0193 (2)	
N1A	0.49224 (15)	0.85415 (11)	0.40764 (8)	0.0186 (2)	
H1A	0.501 (2)	0.8540 (18)	0.3491 (13)	0.029 (4)*	
N2A	−0.00745 (16)	1.24405 (11)	0.22270 (8)	0.0224 (2)	
C1A	0.34768 (17)	0.94707 (12)	0.43635 (8)	0.0166 (2)	
C2A	0.22535 (17)	1.04700 (12)	0.35925 (8)	0.0170 (3)	
C3A	0.03689 (18)	1.07807 (13)	0.36737 (9)	0.0192 (3)	
H3A	−0.015799	1.033733	0.419899	0.023*	
C4A	−0.07252 (19)	1.17503 (13)	0.29726 (9)	0.0216 (3)	
H4A	−0.201170	1.193663	0.302356	0.026*	
C5A	0.1744 (2)	1.21472 (13)	0.21610 (9)	0.0228 (3)	
H5A	0.223073	1.263552	0.164086	0.027*	
C6A	0.29529 (18)	1.11650 (13)	0.28150 (9)	0.0206 (3)	
H6A	0.423490	1.097053	0.273364	0.025*	
C7A	0.63972 (18)	0.76998 (13)	0.46636 (9)	0.0191 (3)	
C8A	0.81592 (19)	0.77125 (14)	0.43946 (10)	0.0227 (3)	
C9A	0.96634 (19)	0.69236 (15)	0.49431 (11)	0.0283 (3)	
C10A	0.9442 (2)	0.61128 (14)	0.57893 (11)	0.0301 (3)	
C11A	0.7709 (2)	0.60855 (14)	0.60733 (10)	0.0283 (3)	
C12A	0.62167 (19)	0.68509 (13)	0.55052 (10)	0.0228 (3)	
F1B	0.95005 (10)	0.46098 (7)	0.88679 (5)	0.02124 (17)	
F2B	1.00723 (11)	0.29582 (8)	0.76949 (5)	0.02752 (19)	
F3B	0.91262 (13)	0.06125 (8)	0.81644 (6)	0.0329 (2)	
F4B	0.76213 (12)	−0.00665 (8)	0.98328 (6)	0.0304 (2)	
F5B	0.70539 (11)	0.15694 (8)	1.10273 (5)	0.02535 (18)	
O1B	0.62254 (12)	0.59168 (9)	0.95399 (6)	0.01814 (19)	
N1B	0.78444 (15)	0.40335 (11)	1.05917 (8)	0.0166 (2)	
H1B	0.831 (2)	0.3691 (18)	1.1126 (12)	0.027 (4)*	
N2B	0.54695 (15)	0.77346 (12)	1.23789 (8)	0.0217 (2)	
C1B	0.68065 (16)	0.53613 (12)	1.03250 (8)	0.0158 (2)	
C2B	0.63837 (17)	0.61433 (13)	1.10735 (9)	0.0169 (2)	
C3B	0.59647 (18)	0.55864 (13)	1.19689 (9)	0.0212 (3)	
H3B	0.599283	0.465630	1.215172	0.025*	
C4B	0.55028 (19)	0.64262 (14)	1.25911 (9)	0.0233 (3)	
H4B	0.519354	0.604944	1.320089	0.028*	
C5B	0.58856 (18)	0.82555 (14)	1.15148 (9)	0.0216 (3)	
H5B	0.588384	0.918061	1.135634	0.026*	
C6B	0.63189 (18)	0.75108 (13)	1.08398 (9)	0.0197 (3)	
H6B	0.656748	0.792679	1.022806	0.024*	
C7B	0.82003 (17)	0.31678 (12)	0.99738 (9)	0.0163 (2)	
C8B	0.89747 (17)	0.34855 (12)	0.91135 (9)	0.0171 (2)	
C9B	0.92841 (18)	0.26397 (13)	0.85043 (9)	0.0200 (3)	
C10B	0.88256 (19)	0.14414 (13)	0.87468 (10)	0.0223 (3)	
C11B	0.80777 (18)	0.10951 (13)	0.95990 (10)	0.0221 (3)	
C12B	0.77824 (17)	0.19421 (13)	1.02067 (9)	0.0190 (3)	

### N-(2,3,4,5,6-Pentafluorophenyl)pyridine-4-carboxamide Atomic displacement parameters (Å^2^)

**Table:**

	*U*^11^	*U*^22^	*U*^33^	*U*^12^	*U*^13^	*U*^23^
F1A	0.0260 (4)	0.0348 (5)	0.0271 (4)	−0.0100 (4)	0.0082 (3)	−0.0137 (4)
F2A	0.0162 (4)	0.0473 (6)	0.0607 (6)	−0.0058 (4)	0.0007 (4)	−0.0239 (5)
F3A	0.0292 (5)	0.0324 (5)	0.0603 (6)	0.0031 (4)	−0.0240 (5)	−0.0077 (4)
F4A	0.0435 (6)	0.0309 (5)	0.0389 (5)	−0.0074 (4)	−0.0159 (4)	0.0116 (4)
F5A	0.0234 (4)	0.0264 (4)	0.0325 (4)	−0.0075 (3)	−0.0027 (3)	0.0031 (3)
O1A	0.0196 (5)	0.0208 (4)	0.0161 (4)	−0.0039 (4)	0.0009 (4)	−0.0047 (3)
N1A	0.0182 (5)	0.0189 (5)	0.0165 (5)	−0.0010 (4)	−0.0017 (4)	−0.0059 (4)
N2A	0.0275 (6)	0.0183 (5)	0.0190 (5)	−0.0035 (5)	−0.0036 (5)	−0.0035 (4)
C1A	0.0167 (6)	0.0159 (6)	0.0180 (6)	−0.0059 (5)	0.0005 (5)	−0.0041 (5)
C2A	0.0199 (6)	0.0150 (6)	0.0164 (6)	−0.0038 (5)	−0.0008 (5)	−0.0059 (5)
C3A	0.0207 (6)	0.0195 (6)	0.0169 (6)	−0.0055 (5)	0.0013 (5)	−0.0044 (5)
C4A	0.0196 (6)	0.0222 (6)	0.0216 (6)	−0.0032 (5)	−0.0012 (5)	−0.0063 (5)
C5A	0.0294 (7)	0.0192 (6)	0.0185 (6)	−0.0075 (5)	0.0017 (5)	−0.0021 (5)
C6A	0.0198 (6)	0.0197 (6)	0.0217 (6)	−0.0055 (5)	0.0019 (5)	−0.0048 (5)
C7A	0.0185 (6)	0.0161 (6)	0.0227 (6)	−0.0011 (5)	−0.0029 (5)	−0.0092 (5)
C8A	0.0221 (7)	0.0209 (6)	0.0266 (7)	−0.0037 (5)	0.0012 (6)	−0.0124 (5)
C9A	0.0165 (7)	0.0273 (7)	0.0446 (9)	−0.0025 (6)	−0.0012 (6)	−0.0201 (6)
C10A	0.0246 (7)	0.0197 (7)	0.0429 (9)	0.0029 (6)	−0.0154 (7)	−0.0101 (6)
C11A	0.0313 (8)	0.0180 (6)	0.0320 (8)	−0.0033 (6)	−0.0098 (6)	−0.0023 (6)
C12A	0.0213 (7)	0.0179 (6)	0.0282 (7)	−0.0033 (5)	−0.0041 (6)	−0.0055 (5)
F1B	0.0208 (4)	0.0165 (4)	0.0267 (4)	−0.0073 (3)	0.0038 (3)	−0.0043 (3)
F2B	0.0306 (5)	0.0267 (4)	0.0200 (4)	−0.0015 (3)	0.0053 (3)	−0.0063 (3)
F3B	0.0402 (5)	0.0251 (4)	0.0367 (5)	−0.0040 (4)	−0.0032 (4)	−0.0201 (4)
F4B	0.0339 (5)	0.0154 (4)	0.0445 (5)	−0.0108 (3)	−0.0024 (4)	−0.0065 (3)
F5B	0.0279 (4)	0.0195 (4)	0.0253 (4)	−0.0076 (3)	0.0034 (3)	0.0008 (3)
O1B	0.0188 (5)	0.0168 (4)	0.0168 (4)	−0.0034 (3)	−0.0016 (4)	−0.0025 (3)
N1B	0.0177 (5)	0.0153 (5)	0.0155 (5)	−0.0026 (4)	−0.0035 (4)	−0.0032 (4)
N2B	0.0185 (6)	0.0239 (6)	0.0226 (6)	−0.0027 (4)	−0.0012 (4)	−0.0098 (4)
C1B	0.0135 (6)	0.0161 (6)	0.0182 (6)	−0.0054 (5)	0.0010 (5)	−0.0039 (5)
C2B	0.0128 (6)	0.0178 (6)	0.0194 (6)	−0.0024 (5)	−0.0022 (5)	−0.0053 (5)
C3B	0.0227 (7)	0.0181 (6)	0.0211 (6)	−0.0046 (5)	0.0005 (5)	−0.0034 (5)
C4B	0.0238 (7)	0.0256 (7)	0.0181 (6)	−0.0044 (5)	0.0012 (5)	−0.0049 (5)
C5B	0.0214 (7)	0.0189 (6)	0.0255 (7)	−0.0056 (5)	−0.0009 (5)	−0.0073 (5)
C6B	0.0201 (6)	0.0188 (6)	0.0194 (6)	−0.0054 (5)	−0.0002 (5)	−0.0036 (5)
C7B	0.0133 (6)	0.0150 (6)	0.0187 (6)	−0.0012 (5)	−0.0037 (5)	−0.0038 (5)
C8B	0.0137 (6)	0.0134 (5)	0.0222 (6)	−0.0022 (5)	−0.0029 (5)	−0.0022 (5)
C9B	0.0175 (6)	0.0199 (6)	0.0187 (6)	0.0001 (5)	−0.0020 (5)	−0.0045 (5)
C10B	0.0215 (7)	0.0184 (6)	0.0264 (7)	0.0000 (5)	−0.0051 (5)	−0.0105 (5)
C11B	0.0201 (7)	0.0127 (6)	0.0326 (7)	−0.0035 (5)	−0.0059 (6)	−0.0041 (5)
C12B	0.0163 (6)	0.0164 (6)	0.0210 (6)	−0.0026 (5)	−0.0024 (5)	−0.0005 (5)

### N-(2,3,4,5,6-Pentafluorophenyl)pyridine-4-carboxamide Geometric parameters (Å, º)

**Table:**

F1A—C8A	1.3351 (16)	F1B—C8B	1.3361 (14)
F2A—C9A	1.3415 (17)	F2B—C9B	1.3351 (15)
F3A—C10A	1.3374 (17)	F3B—C10B	1.3371 (15)
F4A—C11A	1.3381 (18)	F4B—C11B	1.3429 (15)
F5A—C12A	1.3413 (16)	F5B—C12B	1.3382 (15)
O1A—C1A	1.2193 (15)	O1B—C1B	1.2208 (15)
N1A—H1A	0.872 (18)	N1B—H1B	0.844 (18)
N1A—C1A	1.3606 (16)	N1B—C1B	1.3589 (16)
N1A—C7A	1.4047 (17)	N1B—C7B	1.4060 (16)
N2A—C4A	1.3390 (18)	N2B—C4B	1.3367 (18)
N2A—C5A	1.3397 (19)	N2B—C5B	1.3372 (18)
C1A—C2A	1.5046 (18)	C1B—C2B	1.5044 (17)
C2A—C3A	1.3891 (18)	C2B—C3B	1.3875 (18)
C2A—C6A	1.3922 (18)	C2B—C6B	1.3905 (18)
C3A—H3A	0.9500	C3B—H3B	0.9500
C3A—C4A	1.3845 (19)	C3B—C4B	1.3899 (19)
C4A—H4A	0.9500	C4B—H4B	0.9500
C5A—H5A	0.9500	C5B—H5B	0.9500
C5A—C6A	1.3880 (19)	C5B—C6B	1.3847 (18)
C6A—H6A	0.9500	C6B—H6B	0.9500
C7A—C8A	1.3940 (19)	C7B—C8B	1.3928 (18)
C7A—C12A	1.3859 (19)	C7B—C12B	1.3913 (17)
C8A—C9A	1.378 (2)	C8B—C9B	1.3820 (18)
C9A—C10A	1.381 (2)	C9B—C10B	1.3804 (19)
C10A—C11A	1.381 (2)	C10B—C11B	1.379 (2)
C11A—C12A	1.382 (2)	C11B—C12B	1.3821 (19)
			
C1A—N1A—H1A	118.8 (12)	C1B—N1B—H1B	122.4 (12)
C1A—N1A—C7A	122.49 (11)	C1B—N1B—C7B	120.68 (11)
C7A—N1A—H1A	118.2 (12)	C7B—N1B—H1B	116.9 (12)
C4A—N2A—C5A	117.18 (11)	C4B—N2B—C5B	117.51 (11)
O1A—C1A—N1A	124.29 (12)	O1B—C1B—N1B	124.09 (11)
O1A—C1A—C2A	121.61 (11)	O1B—C1B—C2B	120.45 (11)
N1A—C1A—C2A	114.09 (11)	N1B—C1B—C2B	115.46 (11)
C3A—C2A—C1A	119.81 (11)	C3B—C2B—C1B	123.18 (11)
C3A—C2A—C6A	118.34 (12)	C3B—C2B—C6B	118.68 (12)
C6A—C2A—C1A	121.72 (11)	C6B—C2B—C1B	118.05 (11)
C2A—C3A—H3A	120.7	C2B—C3B—H3B	120.9
C4A—C3A—C2A	118.58 (12)	C2B—C3B—C4B	118.19 (12)
C4A—C3A—H3A	120.7	C4B—C3B—H3B	120.9
N2A—C4A—C3A	123.78 (12)	N2B—C4B—C3B	123.63 (12)
N2A—C4A—H4A	118.1	N2B—C4B—H4B	118.2
C3A—C4A—H4A	118.1	C3B—C4B—H4B	118.2
N2A—C5A—H5A	118.4	N2B—C5B—H5B	118.4
N2A—C5A—C6A	123.24 (12)	N2B—C5B—C6B	123.13 (12)
C6A—C5A—H5A	118.4	C6B—C5B—H5B	118.4
C2A—C6A—H6A	120.6	C2B—C6B—H6B	120.6
C5A—C6A—C2A	118.84 (12)	C5B—C6B—C2B	118.82 (12)
C5A—C6A—H6A	120.6	C5B—C6B—H6B	120.6
C8A—C7A—N1A	118.68 (12)	C8B—C7B—N1B	122.34 (11)
C12A—C7A—N1A	124.06 (12)	C12B—C7B—N1B	120.48 (11)
C12A—C7A—C8A	117.26 (13)	C12B—C7B—C8B	117.17 (11)
F1A—C8A—C7A	118.93 (12)	F1B—C8B—C7B	120.18 (11)
F1A—C8A—C9A	119.43 (13)	F1B—C8B—C9B	117.96 (11)
C9A—C8A—C7A	121.63 (13)	C9B—C8B—C7B	121.82 (12)
F2A—C9A—C8A	120.13 (14)	F2B—C9B—C8B	120.22 (12)
F2A—C9A—C10A	119.90 (14)	F2B—C9B—C10B	119.98 (12)
C8A—C9A—C10A	119.96 (13)	C10B—C9B—C8B	119.77 (12)
F3A—C10A—C9A	120.22 (14)	F3B—C10B—C9B	120.28 (12)
F3A—C10A—C11A	120.24 (15)	F3B—C10B—C11B	120.16 (12)
C9A—C10A—C11A	119.54 (14)	C11B—C10B—C9B	119.55 (12)
F4A—C11A—C10A	120.54 (13)	F4B—C11B—C10B	119.57 (12)
F4A—C11A—C12A	119.51 (14)	F4B—C11B—C12B	120.09 (12)
C10A—C11A—C12A	119.94 (14)	C10B—C11B—C12B	120.34 (12)
F5A—C12A—C7A	120.97 (12)	F5B—C12B—C7B	120.16 (11)
F5A—C12A—C11A	117.39 (13)	F5B—C12B—C11B	118.51 (11)
C11A—C12A—C7A	121.62 (13)	C11B—C12B—C7B	121.32 (12)
			
F1A—C8A—C9A—F2A	−1.21 (19)	F1B—C8B—C9B—F2B	−0.54 (18)
F1A—C8A—C9A—C10A	177.32 (12)	F1B—C8B—C9B—C10B	177.51 (11)
F2A—C9A—C10A—F3A	−0.8 (2)	F2B—C9B—C10B—F3B	−1.66 (19)
F2A—C9A—C10A—C11A	179.44 (13)	F2B—C9B—C10B—C11B	177.47 (12)
F3A—C10A—C11A—F4A	0.3 (2)	F3B—C10B—C11B—F4B	−1.27 (19)
F3A—C10A—C11A—C12A	−178.82 (13)	F3B—C10B—C11B—C12B	179.32 (12)
F4A—C11A—C12A—F5A	−3.3 (2)	F4B—C11B—C12B—F5B	0.60 (18)
F4A—C11A—C12A—C7A	178.16 (12)	F4B—C11B—C12B—C7B	−178.40 (11)
O1A—C1A—C2A—C3A	−46.00 (17)	O1B—C1B—C2B—C3B	139.95 (13)
O1A—C1A—C2A—C6A	129.96 (13)	O1B—C1B—C2B—C6B	−36.60 (17)
N1A—C1A—C2A—C3A	135.40 (12)	N1B—C1B—C2B—C3B	−39.79 (17)
N1A—C1A—C2A—C6A	−48.63 (16)	N1B—C1B—C2B—C6B	143.66 (12)
N1A—C7A—C8A—F1A	0.75 (17)	N1B—C7B—C8B—F1B	3.66 (18)
N1A—C7A—C8A—C9A	179.24 (12)	N1B—C7B—C8B—C9B	−178.66 (12)
N1A—C7A—C12A—F5A	4.3 (2)	N1B—C7B—C12B—F5B	−0.70 (18)
N1A—C7A—C12A—C11A	−177.30 (12)	N1B—C7B—C12B—C11B	178.28 (12)
N2A—C5A—C6A—C2A	−1.7 (2)	N2B—C5B—C6B—C2B	−2.1 (2)
C1A—N1A—C7A—C8A	−124.75 (13)	C1B—N1B—C7B—C8B	55.10 (17)
C1A—N1A—C7A—C12A	54.95 (18)	C1B—N1B—C7B—C12B	−124.91 (13)
C1A—C2A—C3A—C4A	177.07 (11)	C1B—C2B—C3B—C4B	−176.66 (12)
C1A—C2A—C6A—C5A	−175.27 (11)	C1B—C2B—C6B—C5B	178.36 (12)
C2A—C3A—C4A—N2A	−2.0 (2)	C2B—C3B—C4B—N2B	−1.2 (2)
C3A—C2A—C6A—C5A	0.75 (18)	C3B—C2B—C6B—C5B	1.66 (19)
C4A—N2A—C5A—C6A	0.85 (19)	C4B—N2B—C5B—C6B	0.8 (2)
C5A—N2A—C4A—C3A	1.03 (19)	C5B—N2B—C4B—C3B	0.8 (2)
C6A—C2A—C3A—C4A	0.97 (18)	C6B—C2B—C3B—C4B	−0.14 (19)
C7A—N1A—C1A—O1A	−11.0 (2)	C7B—N1B—C1B—O1B	−5.13 (18)
C7A—N1A—C1A—C2A	167.53 (11)	C7B—N1B—C1B—C2B	174.60 (11)
C7A—C8A—C9A—F2A	−179.69 (12)	C7B—C8B—C9B—F2B	−178.26 (11)
C7A—C8A—C9A—C10A	−1.2 (2)	C7B—C8B—C9B—C10B	−0.2 (2)
C8A—C7A—C12A—F5A	−176.05 (11)	C8B—C7B—C12B—F5B	179.28 (11)
C8A—C7A—C12A—C11A	2.40 (19)	C8B—C7B—C12B—C11B	−1.74 (19)
C8A—C9A—C10A—F3A	−179.31 (12)	C8B—C9B—C10B—F3B	−179.71 (12)
C8A—C9A—C10A—C11A	0.9 (2)	C8B—C9B—C10B—C11B	−0.6 (2)
C9A—C10A—C11A—F4A	−179.88 (13)	C9B—C10B—C11B—F4B	179.60 (12)
C9A—C10A—C11A—C12A	1.0 (2)	C9B—C10B—C11B—C12B	0.2 (2)
C10A—C11A—C12A—F5A	175.82 (12)	C10B—C11B—C12B—F5B	180.00 (12)
C10A—C11A—C12A—C7A	−2.7 (2)	C10B—C11B—C12B—C7B	1.0 (2)
C12A—C7A—C8A—F1A	−178.96 (11)	C12B—C7B—C8B—F1B	−176.33 (11)
C12A—C7A—C8A—C9A	−0.48 (19)	C12B—C7B—C8B—C9B	1.35 (19)

### N-(2,3,4,5,6-Pentafluorophenyl)pyridine-4-carboxamide Hydrogen-bond geometry (Å, º)

**Table:**

*D*—H···*A*	*D*—H	H···*A*	*D*···*A*	*D*—H···*A*
N1*A*—H1*A*···N2*B*^i^	0.872 (18)	2.004 (19)	2.8261 (16)	156.9 (16)
N1*B*—H1*B*···N2*A*^ii^	0.844 (18)	2.047 (19)	2.8643 (16)	163.1 (17)
C3*A*—H3*A*···O1*A*^iii^	0.95	2.44	3.2075 (17)	138
C5*A*—H5*A*···O1*B*^iv^	0.95	2.54	3.4785 (17)	170
C6*B*—H6*B*···F4*B*^v^	0.95	2.43	3.0669 (16)	124

Symmetry codes: (i) *x*, *y*, *z*−1; (ii) *x*+1, *y*−1, *z*+1; (iii) −*x*, −*y*+2, −*z*+1; (iv) −*x*+1, −*y*+2, −*z*+1; (v) *x*, *y*+1, *z*.
